# *Rhodospirillum* sp. JY3: An innovative tool to mitigate the phytotoxic impact of galaxolide on wheat (*Triticum aestivum*) and faba bean (*Vicia faba*) plants

**DOI:** 10.3389/fpls.2022.1037474

**Published:** 2022-11-14

**Authors:** Mahmoud M. Y. Madnay, Wael A. Obaid, Samy Selim, Ahmed Mohamed Reyad, Emad A. Alsherif, Shereen Magdy Korany, Mohamed Abdel-Mawgoud, Hamada AbdElgawad

**Affiliations:** ^1^ Department of Botany and Microbiology, Faculty of Science, Cairo University, Giza, Egypt; ^2^ Biology Department, College of Science, Taibah University, Al-Madinah Al-Munwarah, Saudi Arabia; ^3^ Department of Clinical Laboratory Sciences, College of Applied Medical Sciences, Jouf University, Sakaka, Saudi Arabia; ^4^ Botany and Microbiology Department, Faculty of Science, Beni-Suef University, Beni‒Suef, Egypt; ^5^ Biology Department, Faculty of Science, Jazan University, Jazan, Saudi Arabia; ^6^ Biology Department, College of Science and Arts at Khulis, University of Jeddah, Riyadh, Saudi Arabia; ^7^ Department of Biology, College of Science, Princess Nourah bint Abdulrahman University, Riyadh, Saudi Arabia; ^8^ Department of Medicinal and Aromatic Plants, Desert Research Centre, Cairo, Egypt; ^9^ Integrated Molecular Plant Physiology Research, Department of Biology, University of Antwerp, Antwerp, Belgium

**Keywords:** galaxolide, *Rhodospirillum* sp. JY 3, wheat, faba beans, antioxidants, detoxification metabolism

## Abstract

To date, several studies have considered the phytotoxic impact of cosmetics and personal care products on crop plants. Nonetheless, data are scarce about the toxic impact of galaxolide [hexahydro-hexamethyl cyclopentabenzopyran (HHCB)] on the growth, physiology, and biochemistry of plants from different functional groups. To this end, the impact of HHCB on biomass, photosynthetic efficiency, antioxidant production, and detoxification metabolism of grass (wheat) and legume (faba bean) plants has been investigated. On the other hand, plant growth-promoting bacteria (PGPB) can be effectively applied to reduce HHCB phytotoxicity. HHCB significantly reduced the biomass accumulation and the photosynthetic machinery of both crops, but to more extent for wheat. This growth reduction was concomitant with induced oxidative damage and decreased antioxidant defense system. To mitigate HHCB toxicity, a bioactive strain of diazotrophic plant growth-promoting *Rhodospirillum* sp. JY3 was isolated from heavy metal-contaminated soil in Jazan, Kingdom of Saudi Arabia, and applied to both crops. Overall, *Rhodospirillum* mitigated HHCB-induced stress by differently modulating the oxidative burst [malondialdehyde (MDA), hydrogen peroxide (H_2_O_2_), and protein oxidation] in both wheat and faba beans. This alleviation was coincident with improvement in plant biomass and photosynthetic efficiency, particularly in wheat crops. Considering the antioxidant defense system, JY3 augmented the antioxidants in both wheat and faba beans and the detoxification metabolism under HHCB stress conditions. More interestingly, inoculation with JY3 further enhanced the tolerance level of both wheat and faba beans against contamination with HHCB *via* quenching the lignin metabolism. Overall, this study advanced our understanding of the physiological and biochemical mechanisms underlying HHCB stress and mitigating its impact using *Rhodospirillum* sp. JY3, which may strikingly reduce the environmental risks on agriculture sustainability.

## Introduction

The excessive use of cosmetics and personal care products has led to the accumulation of contaminants in the environment. Among these contaminants, galaxolide [hexahydro-hexamethyl cyclopentabenzopyran (HHCB)] and tonalide [6-acetyl-1,1,2,4,4,7-hexamethyltetraline (AHTN)] exemplify about 95% of the fragrance compounds used in the perfume industry (Ehiguese et al., 2021). Moreover, HHCB is the most common in various personal care products such as hand soaps, body lotions, laundry detergents, and toothpastes (Schmeiser et al., 2001; An et al., 2009b). In Europe, for instance, people use more than 1,800 tons of HHCB annually, as it is stable and cheap (Balk and Ford, 1999; Bester, 2004). Due to the incessant introduction of HHCB to the environment with no removal strategies, this compound is widely detected in the ecosystem and biological tissues (Díaz-Garduño et al., 2017). In this regard, significant amounts of HHCB are reported in sewage water, surface water, groundwater, soils, and sediments (McAvoy et al., 2002; Singer et al., 2002; Ricking et al., 2003). The levels of HHCB in the ecosystem cannot be overlooked particularly in the sludge in which the levels of HHCB were raised from 6.1 to 61.5 mg/L, the thing that inevitably imperils the health of our ecosystem (Kupper et al., 2004; Shek et al., 2008). In addition to the environment, food safety is also at risk due to the potential impacts of HHCB that in turn affect the public health of humans. To understand the harmful effects of such compounds, the scientific community gave special concern to their effects on aquatic and terrestrial biota. For instance, HHCB and tonalide impose a harmful impact on marine microalgae, invertebrates, and fish (Ehiguese et al., 2021). Moreover, HHCB caused a deleterious effect on the oxidative homeostasis and/or DNA of zebra mussel hemocytes (Binelli et al., 2009). However, little is known about the phytotoxic impact of such compounds on the growth, development, and productivity of economical crops. In this context, wheat growth and development were strikingly retarded by both triclosan and HHCB (An et al., 2009b). Moreover, higher levels of quaternary ammonium compounds including HHCB remarkably retarded the growth of *Triticum aestivum* by reducing shoot and root length and biomass and retarding photosynthetic efficiency and antioxidant homeostasis (Li et al., 2019).

Enhancing crop production deserves to be a major concern of the scientific community to meet human food demands. The intensive usage of agrochemicals strikingly impairs both the ecosystem and public health. To avoid the massive usage of such agrochemicals, several strategies have been used to preserve sustainable agriculture. Among these promising strategies is the use of diazotrophic plant growth-promoting bacteria (PGPB) *via* manipulating their microbiome (Hakim et al., 2021). Diazotrophic PGPB not only enhance plant growth but also alleviate the adverse effects of environmental stresses by augmenting plant stress tolerance and enhancing crop productivity (Kumar and Dubey, 2020). Diazotrophic PGPB can associate with several plant species and enhance their growth to cope with the hazards of environmental cues. These bacteria can augment plant growth either directly or indirectly (Silva Pankievicz et al., 2021). PGPB can directly include the production of plant growth regulators, cell wall- degrading enzymes, and siderophores as well as they can fix nitrogen and produce antibiotics (Gopalakrishnan et al., 2018). They can improve plant growth by enhancing the production of phytohormones and antioxidants and phosphorous fertilization that in turn will reflect on plant physiology and metabolism (Inagaki et al., 2014; Gopalakrishnan et al., 2018; Van Deynze et al., 2018; Mandon et al., 2021; Silva Pankievicz et al., 2021). Furthermore, diazotrophic PGPB improved plant growth under different stressful conditions such as water scarcity (Aguiar et al., 2016; Matoso et al., 2021; Vocciante et al., 2022). Among the diazotrophic PGPB, *Rhodospirillum* sp. is considered as a promising biofertilizer. In this regard, *Rhodospirillum* is widely used in Asia for various biotechnological and agricultural applications such as aquaculture supplements, biofertilizers for plant growth, and bioremediators (Kim et al., 2004). Rana et al. (2016) revealed that *Rhodospirillum rubrum* showed good mineral solubilization and plant growth-promoting activities. Concerning its plant growth-promoting effect, *Rhodospirillum* has bioactive compounds and plant hormones expressing high physiological activities (Serdyuk et al., 1993). Therefore, being environmentally friendly organisms, as they naturally coexist in our ecosystem, *Rhodospirillum* can be used as a biological fertilizer. *Rhodospirillum* application is both environmentally safe and economically inexpensive.

To our knowledge, there is a lack of comprehensive data on the phytotoxic impact of HHCB upon plant species of different functional groups. Therefore, we aimed in this study to investigate whether HHCB implements a phytotoxic impact upon grasses (wheat) and legumes (faba beans). Furthermore, we investigated, for the first time, the hypothesis that changes that improved antioxidant and detoxification systems underlie the HHCB stress-mitigating effect of *Rhodospirillum* sp. JY3. Also, we test the idea that differences between species in plant responses to HHCB stress may be related to differences in redox metabolism.

## Materials and methods

### Isolation and morphological and biochemical characterization of the isolated strain

Rhizosphere soils of *Trianthema portulacastrum* plants were collected from Jazan, Kingdom of Saudi Arabia. The soils were serially diluted and plated on corn meal agar before incubation for 7 days at 40°C. Pale pink to reddish colonies were purified and maintained on nutrient agar. The purified isolate was designated as JY3, and it was preserved on agar slants at 4°C. Morphological properties including cell shape and motility, Gram reaction, mortality, shape, and colony color were determined using light microscopy. Growth and fermentation of carbon sources such as glucose, lactose, and sucrose were tested at 0.1 % (v/v) of growth media.

### Molecular identification

#### Molecular phylogenic identification was done using 16s rDNA amplification

Purification and standard sequencing for PCR products were carried out by Macrogen Company (Seoul, Korea). Sequencing reactions were done by using ABI PRISM^®^ BigDyeTM Terminator Cycle Sequencing Kits with AmpliTaq^®^ DNA polymerase according to the protocols supplied by the manufacturer. The universal primer 27F [5’-AGAGTTTGATC(AC)TGCCTCAG-3’] (forward) primer was used. The fluorescence-labeled fragments were purified according to BigDye^®^ XTerminator™ purification protocol. The sequences were searched for sequence similarity through BLAST (www.ncbi.nlm.nih.gov/BLAST/). The sequences were also compared to reference sequences [GenBank (www.ncbi.nlm.nih.gov/genbank/)].

### Experimental setup and preparation of plant materials

Healthy and uniform seeds of wheat (*T. aestivum* cv. *Giza 119*) and faba beans *(Vicia faba* cv. *Giza 82)* were grown in pots (20 cm × 25 cm) filled with sterilized clay soil (0.5 kg; Tref EGO substrates, Nederland) to remove the other microorganisms. For inoculation, the soils were divided into two groups: 1) treated soil preincubated with 25 ml of log-phase *Rhodospirillum* sp. JY3 culture and 2) control soil that was preincubated with 25 ml of bacterium-free culture medium. Each group of soil was divided into two subgroups: soil treated with galaxolide (HHCB) (250 mg kg^−1^ soil) and untreated soil (control). The HHCB dosage used was chosen after preliminary investigation of different HHCB concentrations (0–500 mg/kg soil) on both seeds of grass and legume. Each of the two plant species were exposed to four treatments, and each treatment comprised three pots, each contains two plants. The three pots represent the three biological replicates per treatment. Plants were grown under controlled conditions in the growth cabinet (21°C/18°C, 16/8 h day/night cycle, 200 μmol Photosynthetic Active Radiation (PAR) m^-2^ s^-1^, and 56% humidity). The soil was watered twice a day and maintained at 58%. Both fresh weight (FW) and dry weight (DW) of shoots were assessed after 7 weeks of growth and stored at -80°C until biochemical analyses.

### Growth rate and photosynthetic measurements

Light-saturated photosynthetic and respiration rates were measured using LI-COR LI-6400 (LI-COR Inc., Lincoln, NE, USA). Stomatal conductance (g_s_) was measured using Leaf Porometer (Model SC1, Pullman, WA, USA). The photochemical efficiency of PSII (Fv/Fm) for dark adapted leaves was calculated with a fluorimeter (PAM2000, Walz, Germany). The concentrations of pigments were assayed (Zhao et al., 2008).

### Determination of oxidative stress markers

Hydrogen peroxide (H_2_O_2_) was measured as described in the methods of de Sousa et al. (2017). Malondialdehyde (MDA) level was determined in 0.1 g frozen tissue according to the method of AbdElgawad et al. (2016) and was calculated as described previously (Hodges et al., 1999). Protein carbonyl colorimetric assay kit was used to measure protein carbonyls (Cayman Chemical, Ann Arbor, MI, USA) (Levine et al., 1994).

### Determination of molecular antioxidants

Total antioxidant capacity was measured by a modified Fe^3+^-reducing antioxidant power (FRAP) assay (de Sousa et al., 2017). Total phenolic content and flavonoids were extracted in ethanol (80%, v/v) and estimated as previously described (de Sousa et al., 2017). Tocopherol (Toco), glutathione (GSH), and ascorbate (ASC) levels were measured by reverse or forward High-performance liquid chromatography (HPLC) methods. For more detail, see de Sousa et al. (2017).

### Determination of reactive oxygen species scavenging enzymes

The antioxidant enzymes, i.e., superoxide dismutase (SOD), ASC peroxidase (APX), peroxidases (POXs), guaiacol peroxidases (GPXs), catalase (CAT), monodehydro-ASC reductase (MDHAR), dehydro-ASC reductase (DHAR), GSH reductase (GR), and GSH-S-transferase (GST), were extracted in 50 mM K-PO_4_ buffer (pH 7.0) containing 10% (w/v) polyvinylpyrrolidone (PVP), 0.25% (v/v) Triton X-100, and 1 mM phenylmethylsulfonyl fluoride (PMSF). The SOD activity was determined by measuring the inhibition of nitro blue tetrazolium (NBT) reduction at 560 nm (de Sousa et al., 2017). APX, MDHAR, DHAR, and GR activities were measured as previously described (Murshed et al., 2008). GPX, GST, and POX activities were determined according to the method of de Sousa et al. (2017), and CAT activity was assayed according to the method of Aebi (1984). Glutaredoxin (Grx) was determined by following the reduction of 2-hydroxy-ethyldisulfide by glutathione in the presence of NADPH and yeast GR and the reduction of NADPH at 340 nm was measured. Thioredoxin (Trx) activity was determined by measuring the reduction of nicotinamide adenine dinucleotide phosphate (NADPH) at 340 (Buchanan et al., 1979). Peroxiredoxin (Prx) activity was determined by measuring the decrease in H_2_O_2_ content according to the method of Horling et al. (2003).

### Detoxification metabolism

Total GSH concentrations were measured by the HPLC method after reduction with dithiothreitol (DTT) (Zinta et al., 2014). GST activity was measured according to the method described by Mozer et al. (1983). Metallothionein-containing protein (MTC) content was measured by using the electrochemical method (Diopan et al., 2008). Total phytochelatins (PCs) and total non-protein thiols in plant samples were extracted in sulfosalicylic acid (5%) and estimated by Ellman’s reagent at 412 nm (De Knecht et al., 1992) by measuring the difference between total non-protein thiols and GSH.

### Lignin content and lignin biosynthetic enzymes

For lignin determination, 0.1 g DW of plant material was homogenized in 95% ethanol and centrifuged at 14,000 g at 4°C for 3 min. After washing with organic solvents at high temperatures, the pellet was mixed with 25% acetyl bromide in acetic acid (1:3, v/v) for 30 min at 70°C. After cooling, NaOH (0.2 ml) and 7.5 M hydroxylamine HCl (0.1 ml) were added and diluted up to 10 ml with acetic acid. After centrifugation at 1,000 g for 4.5 min, the absorbance was measured at 280 nm (Lin and Kao, 2001).

Phenylalanine ammonia lyase (PAL) was extracted in 1 ml of 200 mM sodium borate buffer pH 8.8 according to Koukol and Conn (1961) and assayed by measuring trans-cinnamic acid production at 290 nm. During cinnamyl alcohol dehydrogenase (CAD) analysis, 5 g frozen tissue powder was extracted (Tris : HCl buffer, 200 mM, pH 7.5) (Mansell et al., 1974). For assaying the CAD activity, the formation of coniferyl aldehyde from coniferyl alcohol was measured by the increase in absorbance at 400 nm at 37°C for 5 min. To assay 4-coumarate-coenzyme A ligase (4CL) activity, the method of Voo et al. (1995) was used, involving monitoring the increase in p-coumarate as substrate at 333 nm.

### Statistical analysis

The data presented are the means of three replicates ± standard error (SE). The data were analyzed using SPSS v24.0 (SPSS, Inc.), and significant means were separated using Duncan’s multiple-range tests at P < 0.05.

## Results

### *Rhodospirillum* sp. JY3 inoculation alleviated the adverse effects of HHCB on the biomass and photosynthesis of both faba beans and wheat

**Figure 1:**
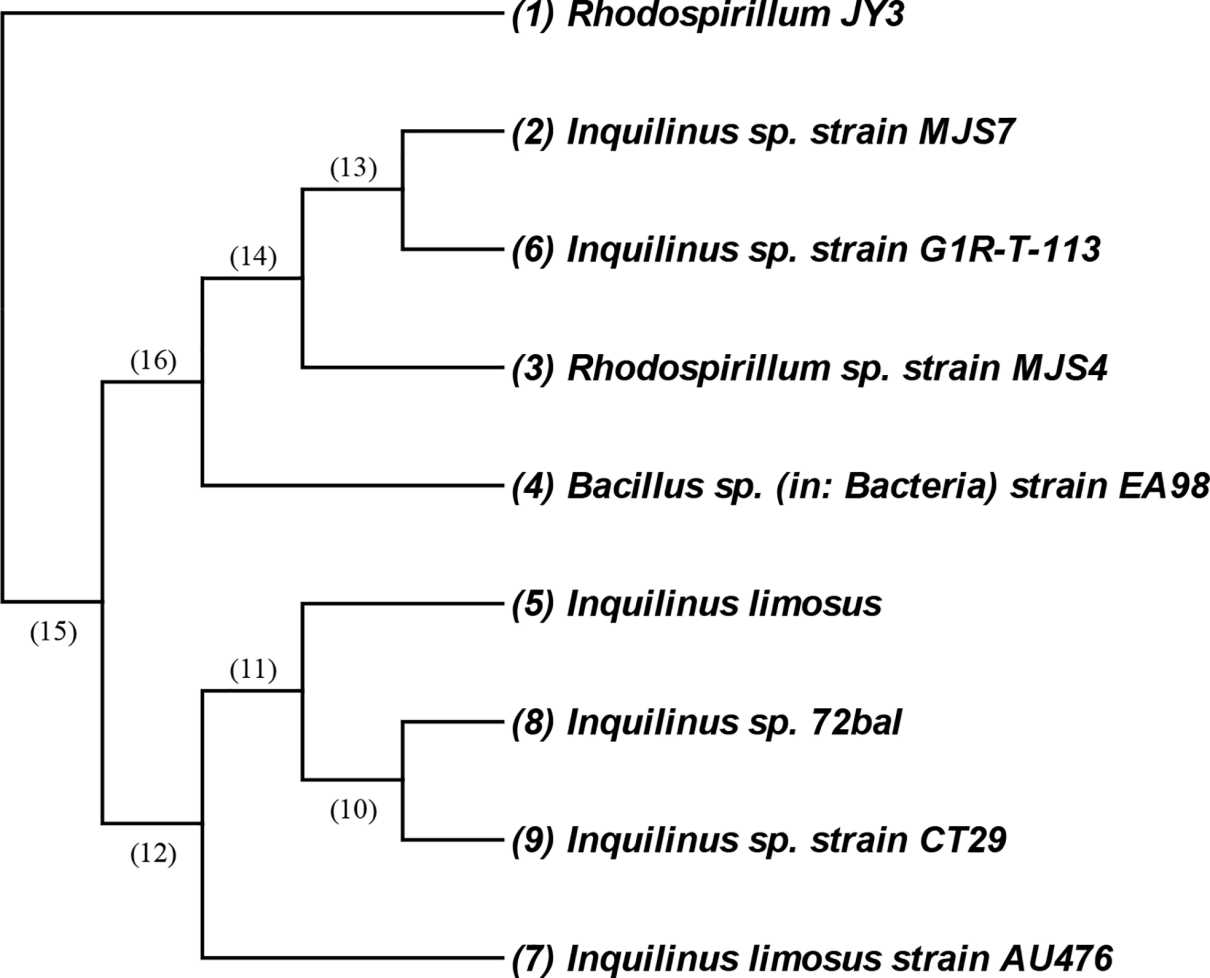
An amplified 16S rRNA gene fragment from the isolated JY3 was sequenced and blast searched through the NCBI database. Closely related sequences were downloaded and aligned using MEGA. The isolate of JY3 was presented in the same clade with *Rhodospirillum* sp. strain MJS4 16S ribosomal RNA gene, partial sequence (90.86%).

The bacterial isolate coded as JY3 was identified morphologically, biochemically, and molecularly by using 16s DNA analysis (**Figure 1**). The cells of this isolate are Gram negative and mobile and give a negative gelatin activity test (**Figure S1**). It showed glucose, lactose, and sucrose fermentation activity. Enzymatic activities revealed that the bacterial isolate JY3 showed amylase, lipase, and protease activities (**Table 1**). According to molecular analyses, gene sequence analysis showed 90.86% similarity with *Rhodospirillum* sp. This bacterial isolate was previously used as a green fertilizer for agriculture. Thus, in our study, we have investigated the ability of this bacterium to improve plant stress tolerance *via* enhancing the growth and biomass production of two different plant species, grasses (wheat) and legumes (faba beans), under control and **HHCB** contamination conditions.

**Table 1:** Morphological and biochemical characterization of selected isolate.

The bacterial test	The result
Gram test	–
Mobility of cell	+
Fermentation	+
Glucose	+
Lactose	+
Sucrose	+
Antifungal activity	+
Production of amylase	+
Protease	+
Lipase	+

(-) or (+) means positivity or negativity of the implement test.

**Figure 2:**
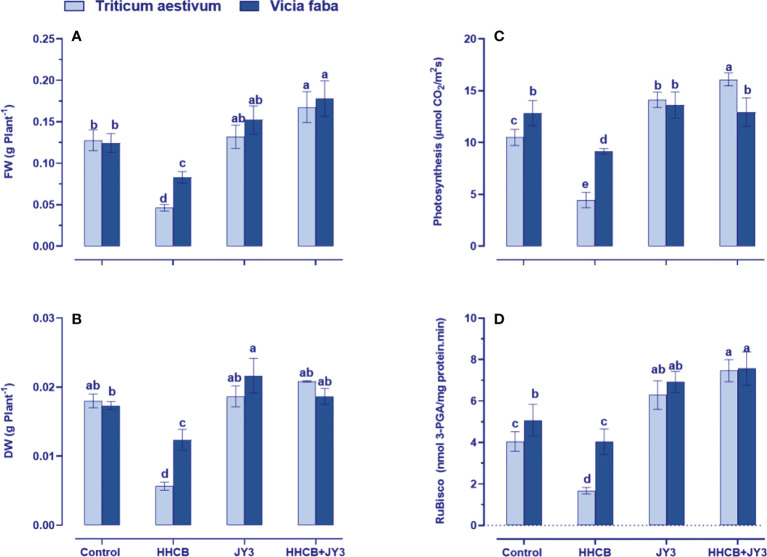
Effect of HHCB (hexahydro-hexamethyl cyclopentabenzopyran) either alone or in combination with Rhodospirillum sp. JY3 on **(A)** Fresh weight, **(B)** Dry weight, **(C)** photosynthesis, and **(D)** RuBisco activiy of both wheat and faba beans. Three biological replicates were used to detect each value. The bars on the columns represent error bars (SE). One-way ANOVA test was use to determine the significant difference between the groups. Similar letters on the bars indicate no significant difference between treatments. Fresh weight (FW), dry weight (DW), ribulose-1,5-bisphosphate carboxylase-oxygnase (RuBisco).

Our findings revealed that treatment with HHCB has resulted in a significant reduction in FW of both wheat and faba beans (63.76% and 33.34% reduction, respectively); meanwhile, a similar reduction was observed in the DW of wheat and faba beans by about 67.28% and 28.38%, respectively (**Figures 2A, B**). On the other hand, treatment with *Rhodospirillum* sp. JY3 slightly enriched the FW and DW of wheat with more increment in those of faba beans as compared with untreated control plants. Interestingly, coapplication of *Rhodospirillum* and HHCB had a flourishment impact on both FW and DW of wheat (~46% for each) and faba beans (~13% and 52%, respectively).

To understand the observed increases in growth, the photosynthetic activity of treated plants was measured (**Figure 2C**). It is known that the photosynthetic activity could be induced by growth-promoting bacterial treatment, while the use of some contaminants such as HHCB could negatively affect photosynthesis (Abdelgawad et al., 2020). In this regard, our results demonstrated that photosynthesis was greatly reduced by HHCB treatment in wheat and faba beans (by about 56% and 33%, respectively) relative to the control plants. Such reductions were recovered when wheat and faba beans were treated with *Rhodospirillum* sp. JY3 as compared with untreated control plants (**Figure 2C**). Under the combined effect of *Rhodospirillum* sp. JY3 and HHCB, both plants showed higher increases in photosynthetic activity, whereas wheat plants have exhibited a dramatic increase (3-fold increase) but to less extent in faba beans (only 44% increase) as compared with contaminated untreated plants.

To further understand the effect by *Rhodospirillum* sp. JY3 and/or HHCB on the photosynthetic activity, we additionally measured the activity of ribulose-1,5-bisphosphate carboxylase-oxygenase (RuBisCO), a key enzyme involved in carbon fixation for sugar production (**Figure 2D**). The results revealed a severe decrease in RuBisCO activity in both wheat and faba beans (~65% and 29% reduction, respectively) relative to their counter control plants. These reductions have been recovered when the two plant species were inoculated with *Rhodospirillum* sp. JY3 where the activity of RuBisCO increased by 26% and 33%, respectively) in comparison to the untreated control plants. Furthermore, the coapplication of *Rhodospirillum* sp. JY3 and HHCB resulted in higher increments in RuBisCO activity, particularly in wheat (~73% increase), when compared with the control plants. Thus, improving the photosynthetic machinery considered one of the key roles of *Rhodospirillum* sp. JY3 that increased the tolerance level of both wheat and faba beans against contamination with HHCB.

### Inoculation with *Rhodospirillum* sp. JY3 reduced the oxidative damage caused by HHCB

**Figure 3:**
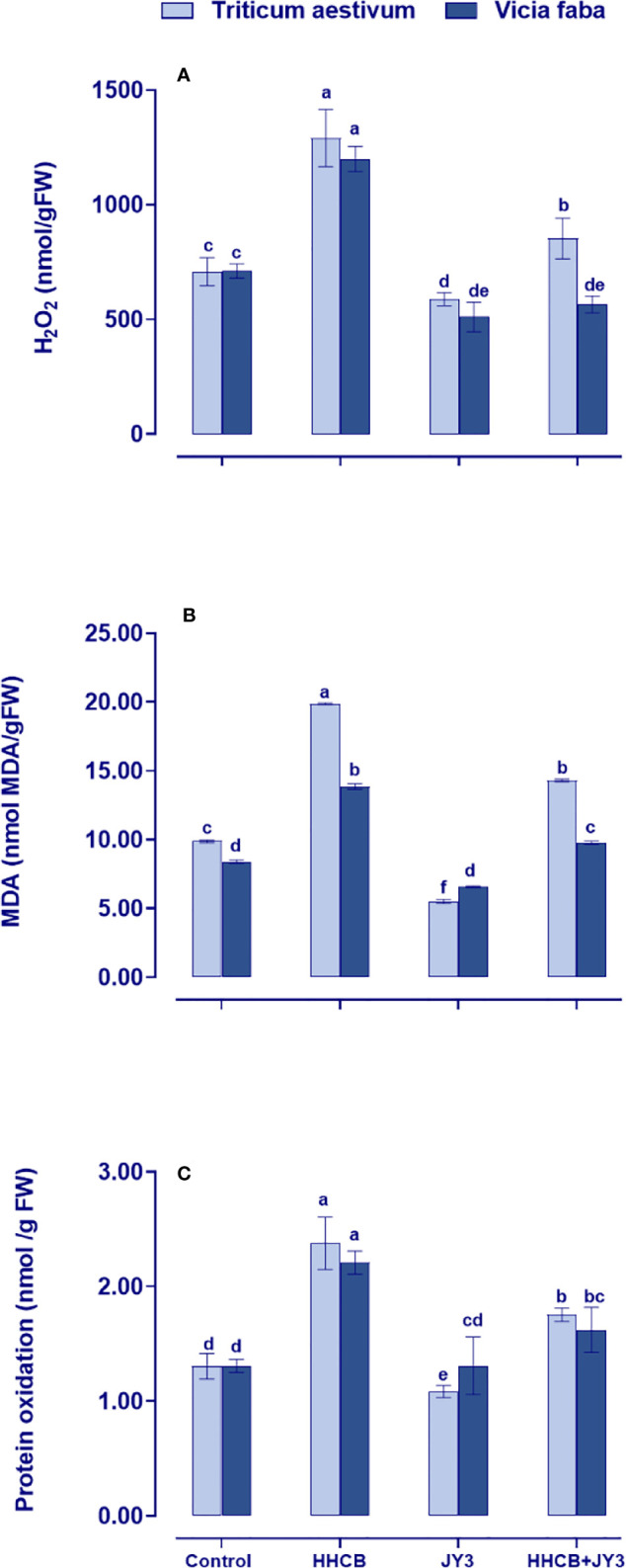
Effect of HHCB either alone or in combination with Rhodospirillum sp. JY3 on the oxidative markers; **(A)** Hydrogen peroxide (H2O2), **(B)** Monoaldehyde (MDA) and **(C)** Protein oxidation of both wheat and faba beans. Three biological replicates were used to detect each value. The bars on the columns represent error bars (SE). One-way ANOVA test was used to determine the significant difference between the groups. Similar letters on the bars indicate no significant difference between treatments.

Under stress conditions, plants might be subjected to higher levels of reactive oxygen species (ROS), which consequently might result in the destruction of membrane lipids, leading to lipid peroxidation. This could induce MDA and protein oxidation that are also associated with H_2_O_2_ generation. In the present study, the changes in oxidative stress markers of wheat and faba beans grown under different treatments with *Rhodospirillum* sp. JY3 and/or HHCB have been investigated (**Figure 3**). Single treatment with HHCB induced significant increases in the amounts of H_2_O_2_ and MDA, as well as protein oxidation in faba beans with much more increment in wheat plants when compared with the untreated control plants. These differential responses to HHCB indicated a species-specific response to oxidative damage. On the other hand, the oxidative damage was significantly ameliorated when both species under investigation were treated with *Rhodospirillum* sp. JY3 (**Figure 3**). Interestingly, the mitigative impact of *Rhodospirillum* sp. JY3 on HHCB has been positively reflected on the noticeable reduction in the levels of oxidative markers (H_2_O_2_, MDA, and protein oxidation) in wheat and faba beans either alone or in combination with HHCB as compared with their respective control plants (**Figure 3**).

### Interactive effect of HHCB and *Rhodospirillum* sp. JY3 on the levels of antioxidant metabolites in both faba beans and wheat

**Figure 4:**
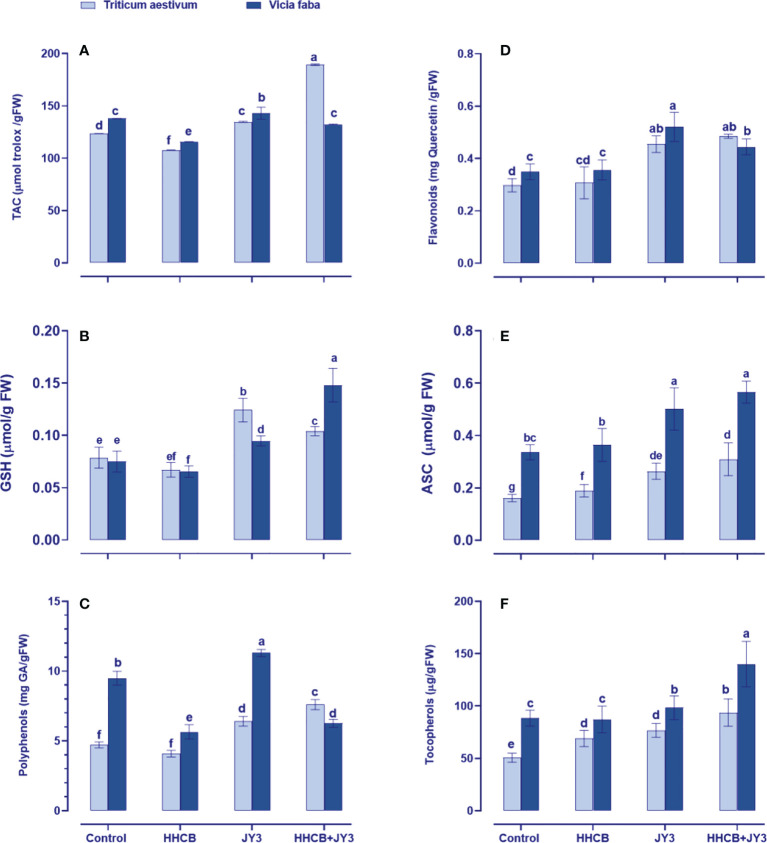
Effect of HHCB either alone or in combination with *Rhodospirillum* sp. JY3 on **(A)** the total antioxidant capacity (TAC), **(B)** glutathione (GSH), **(C)** polyphenols, **(D)** flavonoids, **(E)** ascorbate (ASC), and **(F)** tocopherols of both wheat and faba beans. three biological replicates were used to detect each value. the bars on the columns represent error bars(SE). One-way ANOVA test was used to determine the significant difference between the groups. Similar letters in the bars indicate no significant diffidence between treatments.

In order to mitigate oxidative damages under stress factors, plants tend to upregulate their defense system (Shabbaj et al., 2022). To this end, the wide array of non-enzymatic antioxidants was measured in wheat and faba beans (**Figure 4**). The antioxidant defense system could be detected through the total antioxidant capacity (TAC); thus, we measured TAC by using the FRAP assay (**Figure 4A**). When both plants were treated with HHCB, a species-specific response was observed in TAC, as it significantly reduced in wheat (~13% reduction) with much more reduction in faba beans (~16% reduction) as compared with their counter control plants. On the other hand, *Rhodospirillum* sp. JY3 significantly enhanced TAC in wheat and faba beans. *Rhodospirillum* sp. JY3 further increased the effect of HHCB on TAC, particularly in wheat and to less extent in faba beans (by 37.59% and 14.32%, respectively).

Furthermore, to understand the mechanism behind the changes in TAC, we evaluated the changes in individual antioxidants, either the soluble antioxidants, i.e., flavonoids, polyphenol, GSH, and ASC, or the insoluble antioxidants, i.e., Toco (**Figure 4**). In this context, HHCB has no significant influence on the measured molecular antioxidants except for Toco in wheat that accumulated by about 37% and polyphenols and GSH in faba beans that decreased by 38% and 25%, respectively, as compared with uncontaminated, uninoculated control plants (**Figures 4B, C**). On the other hand, treatment of wheat and faba beans with *Rhodospirillum* sp. JY3 alleviated the adverse effects of HHCB by increasing the levels of flavonoids, polyphenols, and Toco when applied either alone or in combination with HHCB relative to their counter control plants. It is worth observing that Toco accumulation was noticeable in faba beans and wheat plants as compared with contaminated plants grown in Bactria free soils (61% and 35%, respectively), the thing that highlights a species-specific response to bacterial treatment. Except for ASC in faba beans, significant increases were also observed in GSH levels of both wheat and faba beans and ASC only in wheat plants after treatment with *Rhodospirillum* that grew in soils contaminated with HHCB (**Figures 4B, E**). A further increment was observed in both ASC and GSH of both wheat and faba beans. Meanwhile, the interaction between HHCB or *Rhodospirillum* sp. JY3 increased the GSH content of wheat and faba beans (32.33% and 97.29%, respectively) but decreased the ASC content in both plants. Also, comparable increases in the soluble antioxidants (Toco content) were also observed in both plants under all of the single and combined treatments with HHCB and *Rhodospirillum* sp. JY3.

### Coinoculation with *Rhodospirillum* sp. JY3 differently improved antioxidant enzyme activities, particularly under the challenge of galaxolide contamination

**Figure 5:**
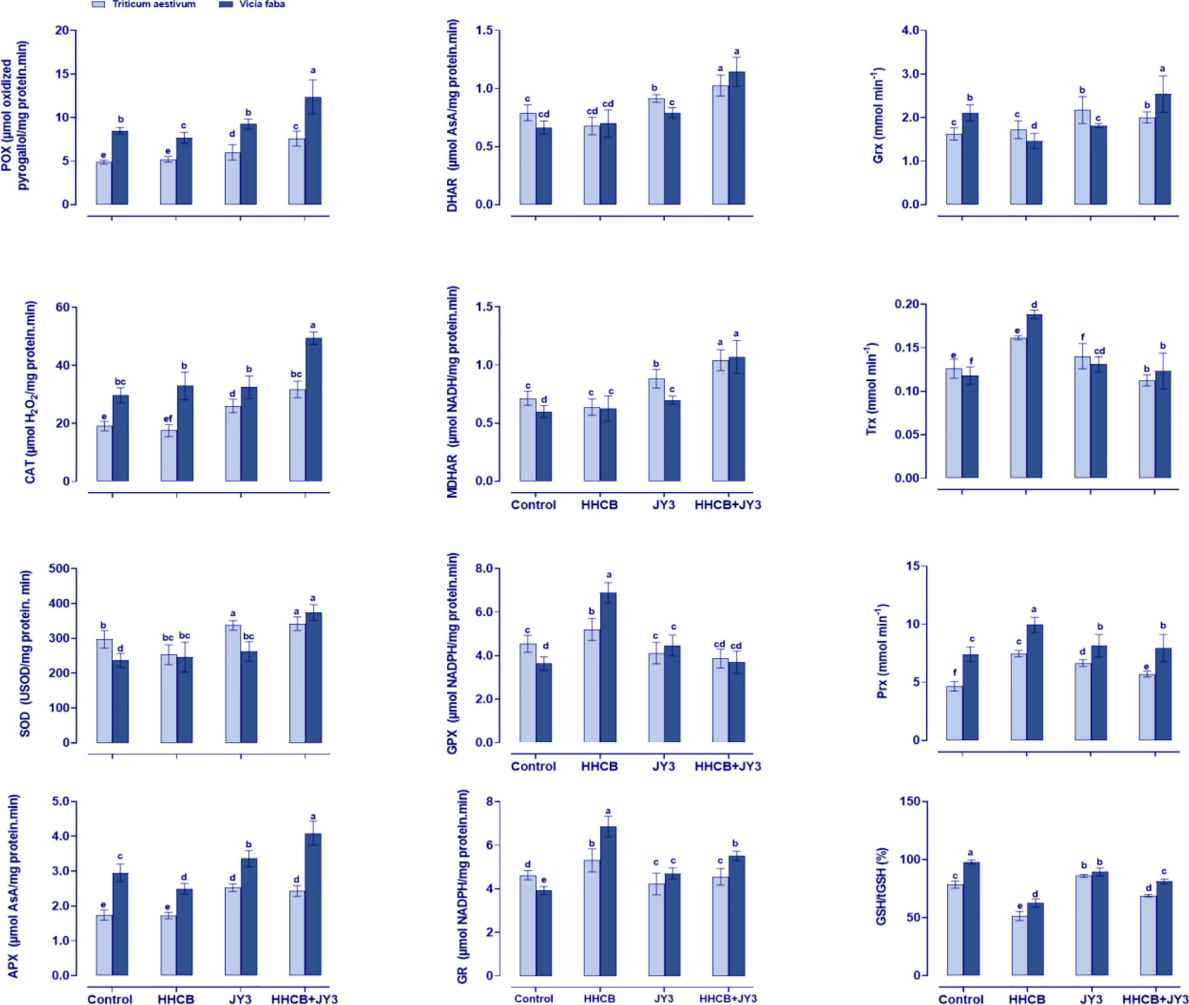
Effect of HHCB either alone or in combination with *Rhodospirillum* sp. JY3 on the antioxidant defense system of both wheat and faba beans. Three biological replicates were used to detect each value. The bars on the columns represent error bars (SE). One-way ANOVA test was used to determine the significant difference between the groups. Similar letters on the bars indicate no significant difference between treatments. SOD; Superoxide dismutase, APX; ascrobate peroxidase, POX; peroxidases, GPX; guaiacol peroxidases, CAT; catalase, MDHAR; monodehydro-ASC reductase, DHAR; dehydro ASC reductase, GR; glutathione reductase, and GST; glutathione-S-transferase, Grx: Glutaredoxin, Trx; thioredoxin and Prx; Peroxiredoxin.

In order to understand how *Rhodospirillum* sp. JY3 reduced the stress induced by HHCB, it was essential to investigate the changes that occurred in the activities of antioxidant enzymes (**Figure 5**). Therefore, we measured the enzymes that had direct scavenging activity. The current results showed that HHCB treatment almost decreased or did not change the POX, CAT, and SOD activities in wheat, while the activity of SOD was slightly increased in faba beans as compared with the control plants. Meanwhile, treatment with *Rhodospirillum* caused remarkable increments in the activities of POX, CAT, and SOD in both wheat and faba beans relative to control plants. Further increments in POX, CAT, and SOD were observed when using a combination of HHCB and *Rhodospirillum* sp. JY3 (by 46.42%, 42.59%, and 35.20%, respectively, in wheat and by 61.39%, 50.18%, and 51.85%, respectively, in faba beans) as compared with uninoculated contaminated control plants.

Concomitantly, we also measured the ASC-related enzymes in the ASC-GSH cycle, i.e., APX that reduced H_2_O_2_ to water by using ASC and the enzyme DHAR that plays a role in the reduction of dehydroascorbate through using GSH. In addition, MDHAR is known to be incorporated in the reduction of monodehydro-ASC. According to our results, treatment of both plants with HHCB reduced or did not change the activities of APX and DHAR, while MDHAR activity was significantly enhanced in wheat and faba beans (37.96% and 64.05%, respectively) when compared with the control plants (**Figure 5**). Likewise, the activities of all of the detected enzymes were enhanced in response to inoculation with *Rhodospirillum* sp. JY3. Moreover, the interaction between HHCB and *Rhodospirillum* sp. JY3 triggered the activities of APX and DHAR in both wheat (~40.63% and 51.72%, respectively) and faba beans (~63.89% and 63.75%, respectively) as compared with control plants (**Figure 5**).

Furthermore, the activities of GPX and GR were investigated, whereas both enzymes play a crucial role in ASC-GSH cycle. Under HHCB stress, the activities of GR and GPX were increased by 14.66% in wheat and by 74.92% and 89.53% in faba beans, respectively. The inoculation with *Rhodospirillum* sp. JY3 significantly increased GR and GPX activities in faba beans (19.73% and 22.95%, respectively) but decreased these activities in wheat plants. The interactive impact of HHCB and *Rhodospirillum* sp. JY3 has been negatively reflected on GR and GPX activities in both plants.

The response of the antioxidant defense system to the impact of HHCB and/or *Rhodospirillum* sp. JY3 upon the enzymes’ activity was further investigated by measuring the activities of Grx, Trx, and Prx in HHCB-stressed plants (**Figure 5**). These enzymes incorporated in scavenging of free radicals that are involved in the reduction of H_2_O_2_. Except for Grx, single treatment with HHCB or *Rhodospirillum* sp. JY3 increased the activities of Trx and Prx in both plant species when compared with the control plants. On the other hand, coapplication of HHCB and *Rhodospirillum* sp. JY3 caused a significant increment in Grx, Trx, and Prx in both plant species under investigation as compared with the control samples.

### Detoxification metabolism was increased in wheat and faba beans in response to the inoculation with *Rhodospirillum* sp. JY3 under HHCB stress

**Figure 6:**
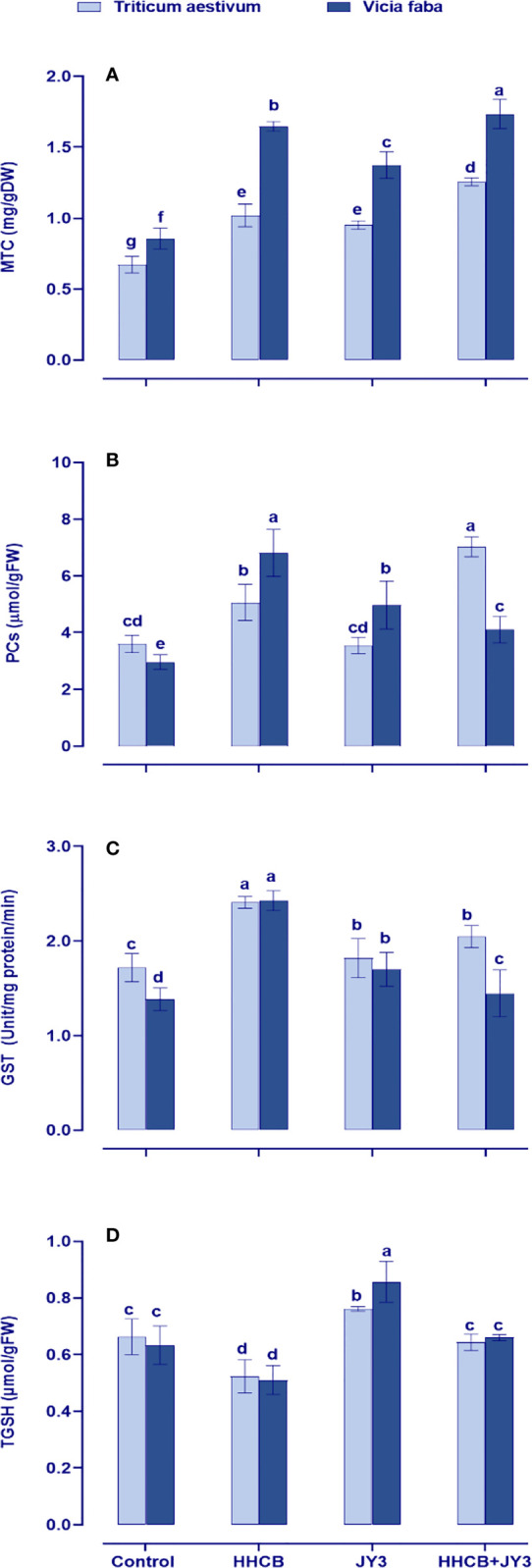
Effect of HHCB either alone or in combination with *Rhodospirillum* sp. JY3 on **(A)** metallothionein (MTC), **(B)** phytochelatins (PCs), **(C)** glutathione-s-transferase (GSH), **(D)** total glutathione (TGSH) of both wheat and faba beans. three biological replicates were used to detect each value. the bars on the columns represent error bars(SE). One-way ANOVA test was used to determine the significant difference between the groups. Similar letters in the bars indicate no significant diffidence between treatments.

In our study, we also evaluated heavy metal-binding ligands, such as metallothioneins (MTCs), PCs, and the metal-detoxifying enzyme GST, which are synthesized by the plant to overcome heavy metal toxicity (**Figure 6**). Under treatment with HHCB, all of the measured parameters were significantly increased in both wheat and faba beans, particularly PCs in faba beans (about 2-fold increase). It is worth noting that individual treatment with *Rhodospirillum* sp. did not affect the levels of such parameters as compared with their counter control plants (**Figure 6**). However, under HHCB treatment, *Rhodospirillum* sp. JY3 caused a significant enhancement in the contents of PCs and MTCs in wheat plants (98% and 32%, respectively) and in faba beans (13% and 26%, respectively), while the activity of GST was reduced in both plants when compared with the control plants.

Moreover, the redox status of GSH (tGSH) was concomitantly retarded in both wheat and faba beans under contamination conditions (**Figure 6D**). This retardation had been enhanced in both plant species when inoculated with *Rhodospirillum* sp. as compared with uncontaminated control plants. Furthermore, inoculation with *Rhodospirillum* alleviated the phytotoxic impact of HHCB by accumulating the levels of tGSH as compared with contaminated uninoculated control plants (**Figure 6D**).

### Lignin metabolism was increased in HHCB-stressed wheat and faba beans in response to *Rhodospirillum* sp. JY3 inoculation

**Figure 7:**
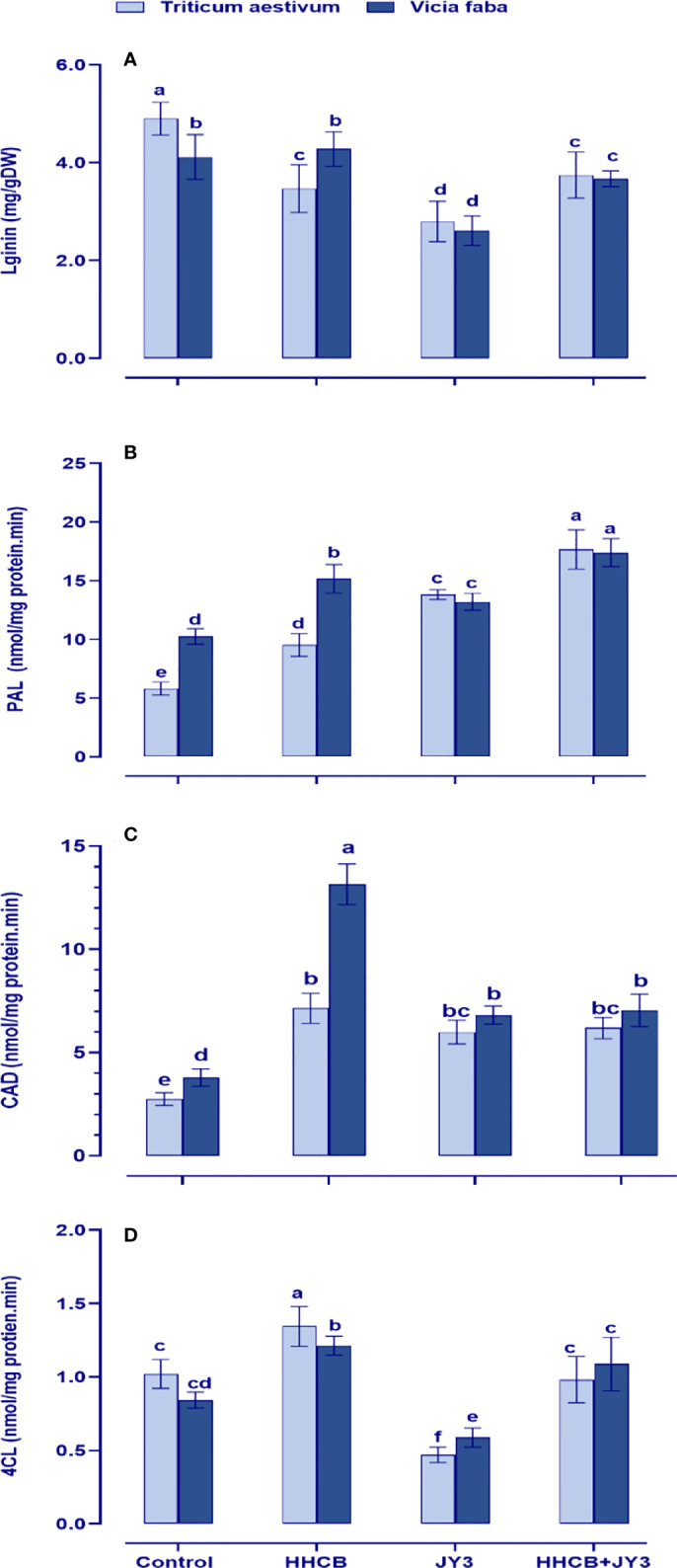
Effect of HHCB either alone or in combination with *Rhodospirillum* sp. JY3 on **(A)** lignin, **(B)** phenylalnine ammonia lyase (PAL), **(C)** cinnamyl alcohol dehydrogenase (CAD), **(D)** 4-coumarate-coenzyme A ligase (4CL) of both wheat and faba beans. three biological replicates were used to detect each value. the bars on the columns represent error bars(SE). One-way ANOVA test was used to determine the significant difference between the groups. Similar letters in the bars indicate no significant diffidence between treatments.

Lignin plays a fundamental sustaining and protective role in plants to cope with various environmental anomalies. Therefore, we interestingly investigated the levels and the biosynthetic enzymes of lignin in both wheat and faba beans (**Figure 7**). Except for the activities of PAL in faba beans, contamination with HHCB significantly reduced the activities of lignin biosynthetic enzymes and accordingly lignin biosynthesis in both wheat and faba beans as compared with their respective untreated control plants. On the other hand, inoculation with *Rhodospirillum* sp. tremendously augmented the accumulation of lignin by activating its biosynthetic enzymes (4CL, CAT, and PAL). Interestingly, coapplication of *Rhodospirillum* sp. with HHCB caused further increment in the activities of 4CL, PAL, and CAD.

## Discussion

HHCB compounds are widely detected in our environment and biological tissues. These accumulations in the ecosystem cannot be ignored, especially in water, as this will impair the health of the environment. Therefore, our objective in this study is to understand the impacts of the environmentally pertinent concentrations of HHCB on the growth as well as antioxidant homeostasis and detoxification mechanism of two economically important crops (wheat and faba beans). Moreover, we applied *Rhodospirillum* sp. JY3 as an environmentally safe tool to mitigate the harmful impact of HHCB on both wheat and faba beans.

### *Rhodospirillum* sp. JY3 greatly mitigated the phytotoxic effect of HHCB on the growth and photosynthesis of both wheat and faba beans

Our results revealed that HHCB caused a noticeable reduction in the growth and biomass of both wheat and faba beans. It is worth mentioning that the personal care products including HHCB are considered as harmful compounds on plant growth and development. In this context, personal care products can implement a significant phytotoxic effect including retardation in their growth and development (Christou et al., 2016; Bartrons and Peñuelas, 2017; Sun et al., 2018). These findings suggest that personal care products such as HHCB can implement retardational effects on plant growth and development including vascular tissue development (Barooah et al., 2022) and cell cycle (DNA replication) (Grzesiuk et al., 2018). As the photosynthetic efficiency of plants is considered one of the most crucial determinants of plant growth, the retarded photosynthetic efficiency of HHCB-stressed plants could be another explanation of the decline in the growth and biomass. In line with our findings, *T. aestivum* seedlings showed a striking inhibition in the photosynthetic efficiency in response to treatment with both HHCB and triclosan (An et al., 2009b). Additionally, cucumber seedlings experienced a remarkable reduction in the biosynthesis of photosynthetic pigments when treated with different pharmaceuticals and personal care products (Sun et al., 2018). Personal care products including HHCB can also affect photosynthetic processes such as the Calvin cycle and DNA replication (Grzesiuk et al., 2018). On the other hand, treatment with *Rhodospirillum* sp. JY3 caused a significant improvement in the biomass of wheat and faba beans either alone or in combination with HHCB. Besides having a characteristic plant growth-promoting activity, *Rhodospirillum* sp. JY3 is the best biofertilizer that has the ability to induce the highest Vigor Index (990) value (Vareeket and Soytong, 2020). *Rhodospirillum* sp. JY3 can also produce plant hormones as bioactive compounds that expressed high physiological activities (300%–330%) in the cytokinin bioassay (Smith et al., 2008). Moreover, *Rhodospirillum* sp. JY3 has a high ability to solubilize minerals that consequentially increases nutrient availability (Vareeket and Soytong, 2020). Therefore, these findings in addition to our results could explain the mitigating impact of *Rhodospirillum* sp. JY3 against HHCB toxicity.

### *Rhodospirillum* sp. differentially mitigated the phytotoxic impact of HHCB *via* relieving the oxidative damage in wheat and faba beans

It is well known that the overproduction of ROS leads to cell damage and is the final concern of oxidative stress. Our results revealed a differential accumulation of ROS in both wheat and faba beans that is embodied in the elevation of both protein oxidation and lipid peroxidation in response to HHCB treatment (**Figure 3**). On the other hand, it is worth noting that the activities of most antioxidant enzymes either were not changed or became low in both wheat and faba beans that might be due to the hormesis effect of HHCB on the plant, the thing that could mitigate the oxidative stress induced by HHCB (Ehiguese et al., 2021). This negative impact of HHCB on antioxidant enzymes, especially POX, SOD, and CAT, could explain the obvious increments in the levels of H_2_O_2_. Therefore, by comparing the results of the biomass and oxidative markers (H_2_O_2_, protein oxidation, and MDA) as well as the activities of antioxidant enzymes, we can find that both wheat and faba beans cannot withstand the adverse effects of HHCB for long periods. This is because the level of HHCB-induced oxidative stress may exceed the capacity of the antioxidant enzymatic pool. This different action indicated a species-specific response toward contamination with HHCB. In line with our findings, Christou et al. (2016) showed that alfalfa leaves exposed to treatment with pharmaceutical products experienced a noticeable lipid peroxidation. To a lesser extent, treatment with different pharmaceutical and personal care products showed a remarkable elevation in lipid peroxidation with a concomitant increase in the activities of SOD that in turn caused noticeable oxidative damage in cucumber plants (Sun et al., 2018). Additionally, the authors found that the root of cucumber exhibited a remarkable oxidative damage with a concomitant elevation in the activities of SOD due to the treatment with pharmaceutical and personal care products. Moreover, treatment of wheat seedlings with quaternary ammonium salts caused an obvious triggering for oxidative damage and lipid peroxidation (Li et al., 2019). Other studies reported that treatment with water pollutants at high levels caused a remarkable increment in their MDA levels in many economically important crops (Cheng, 2012; Liu et al., 2013; Pawłowska et al., 2017). On the other hand, SOD activity significantly decreased due to treatment with triclosan and HHCB (An et al., 2009b), while it increased as a result of treatment with paracetamol in wheat seedlings (An et al., 2009a). In line with these findings, the activities of antioxidant enzymes were strikingly increased with a concomitant increase in both O2^⋅-^ and H_2_O_2_ levels in wheat plants treated with different types of quaternary ammonium compounds (Li et al., 2019). It is worth mentioning that lipid peroxidation caused by such contaminants can further accelerate cell apoptosis, the main cause of cell damage (Wang et al., 2021). Moreover, antioxidants (e.g., SOD, POX, and the enzymatic system of the ASC/GSH cycle) are engaged to trap the overproduced ROS (Shabbaj et al., 2022). Therefore, the changes in the activities of the antioxidant enzymes indicate oxidative stress. Overall, our findings suggested that exposure to HHCB can trigger responses concerning the antioxidants and redox homeostasis and make the plants fail to scavenge the overproduced ROS (**Figures 2**, **4**).

### How *Rhodospirillum* sp. JY3 reduces HHCB-induced oxidative stress by augmenting the antioxidant enzymes

As an environmentally safe tool, treatment with *Rhodospirillum* sp. JY3 strikingly alleviated the adverse impact of HHCB on both wheat and faba beans. The mitigative impact appeared as enhancing the antioxidant defense arsenal. ASC, for instance, can be used by the cell as a reductant that can reduce H_2_O_2_ into H_2_O, a reaction that was catalyzed by APX that was considered as the key step in the ASC/GSH cycle (Madany et al., 2020). Therefore, the elevation in the activity of APX in both wheat and faba beans under the combined effect of both HHCB and bacterial treatment may be attributed to the functioning of ASC-GSH cycle in detoxifying H_2_O_2_ and thus preventing more damage (Paradiso et al., 2008). In addition to APX, CAT has the ability to scavenge the excess ROS induced by stress (Sun et al., 2018). Therefore, the noticeable enhancement in the activity of CAT in wheat and faba beans treated with bacteria reaffirmed that *Rhodospirillum* sp. JY3 can enhance their antioxidant homeostasis to cope with the adverse effects of HHCB. Besides APX and CAT, POX and GST were other key antioxidant enzymes that can scavenge toxic ROS compounds (Karuppanapandian et al., 2011). The enhancement in the activity of such enzymes due to treatment with *Rhodospirillum* sp. JY3 highlights its ability to trigger the detoxifying agents that can detoxify HHCB. Interestingly, it was found that POX can degrade 2,4-dicholophenol in the cell culture of *Brassica napus* (Agostini et al., 2003). Additionally, Xia et al. (2009) implied that the chlorpyrifos-induced activity of GST was accompanied by the formation of glutathione (GSH) S-conjugates to degrade the plant’s insecticide. Moreover, diclofenac was oxidized by oxidases and GSH conjugation in *Typha latifolia* (Huber et al., 2016). In addition to our results, the aforementioned studies clearly suggested that both POX and GST enzymes play a crucial role in the transformation and conjugation of HHCB in the crop plants. Besides being a mandatory antioxidant in plant cells, GSH can provide a common pathway for plants to detoxify environmental pollutants *via* conjugation with xenobiotics (Neuefeind et al., 1997). This process consumes excessive amounts of reduced GSH that acts as an electron donor that in turn leads to the overproduction of oxidized GSH (GSSG) to help in the detoxification of xenobiotic compounds (Sun et al., 2018). These variations in GSH levels, particularly the estimated ratio of reduced to oxidized (GSH/tGSH), makes it play a pivotal role in plant tolerance responses (Shabbaj et al., 2022). In this regard, Zhang et al. (2017) gave a detailed account on the role of thiols such as GSH in the detoxification of atrazine in higher plants. Recently, it was reported that GSH triggered the detoxification and promoted the metabolism of the residual fungicide chlorothalonil (CHT) *via* augmenting UDP-glycosyltransferase genes in tomato plants (Yu et al., 2022a). Additionally, the ASC-GSH pathway not only played a pivotal role in detoxifying CHT residue *via* nitric oxide signaling but also enhanced the gene expression of antioxidant metabolites, promoting the detoxification-related enzymes in tomato plants (Yu et al., 2022b). Therefore, if we consider the observed prevalence of GSH conjugates in pesticides–plant interaction, it is likely that contaminants like HHCB could be similarly detoxified by GSH conjugation.

### *Rhodospirillum* sp. JY3 quenched the detoxification system of both wheat and faba beans to cope with the challenge of HHCB

Another route to enhance the GSH detoxification pathway is the involvement of detoxifying metabolites such as melatonin, PCs, and Grx. In this study, the detoxification metabolism was triggered in response to treatment with HHCB in both wheat and faba beans with further enhancement when treated with *Rhodospirillum* sp. JY3. This improvement in the detoxification metabolism in the presence of these environmental threats is considered as a good strategy that makes the plant able to cope with these cues. In this context, melatonin can alleviate the oxidative damage in cucumber triggered by imidacloprid, a leaf-spraying insecticide (Liu et al., 2021). Moreover, Zimeri et al. (2005) suggested that MTC was essential for Cd tolerance and detoxification in *Arabidopsis thaliana*. More interestingly, the accumulation of metal-binding PCs was detected in cell suspension cultures of *Rauvolfia serpentina* and *Arabidopsis* seedlings in response to arsenic pollution (Schmoger et al., 2000). Additionally, it was reported that GST plays a pivotal role in arsenic detoxification in different plant species (Kumar and Trivedi, 2018). Recently, we found that elevated CO_2_ greatly enhanced the accumulation of detoxifying metabolites such as PCs, MTC, GSH, and TGSH in both C3 and C4 plants to cope with the threat of contamination with indium oxide nanoparticles (Shabbaj et al., 2022). These findings revealed the regulatory role of such detoxifying metabolites in regulating the ROS homeostasis in plants under the challenge of environmental cues. It is worth mentioning that the metal-binding protein MTC regulates the transport of plant metals, while GST manipulates the GSH-metal conjugation (Kumar and Trivedi, 2018). In addition, the cumulation of PCs and GSH oligomers will aid in binding water pollutants and insulate them to the vacuole (Sharma et al., 2016). Overall, our findings highlight the role of *Rhodospirillum* sp. JY3 in enhancing the detoxifying systems in both wheat and faba beans to cope with the challenge of HHCB water contamination.

### *Rhodospirillum* sp. JY3 and lignin metabolism in wheat and faba beans under treatment with HHCB

In addition to the detoxifying system, water contaminants involved metabolic alterations in plant systems such as lignin metabolism. This will in turn disturb lignin as a bioindicator and an efficient line of defense against environmental anomalies. These environmental cues induce the phenylpropanoid pathways by triggering the signaling network *via* extracellular ATP (eATP) and dinucleotide polyphosphates to form several metabolites including lignin (Bhardwaj et al., 2014). Our results revealed an increment in the levels of lignin and its biosynthetic enzymes in response to treatment with HHCB with further increment on treatment with *Rhodospirillum* sp. JY3. The ameliorative role of *Rhodospirillum* sp. JY3 could be attributed to its potentiality to trigger the pathway of phenolic biosynthesis by regulating the levels of H_2_O_2_ and APX under the challenge of HHCB, resulting in the accumulation of lignin in both wheat and faba beans. In this context, the biosynthesis of extracellular lignin and flavonoids was triggered by H_2_O_2_ signal transduction (Rao and Dixon, 2018). Moreover, the formation of monolignol radicals was regulated by APX to detoxify H_2_O_2_, indicating a possible correlation between lignin biosynthesis and H_2_O_2_ biosynthesis (Gou et al., 2018).

## Conclusion

Overall, our study provides a new insight about the adverse impact of HHCB on the growth, photosynthesis, and the antioxidant homeostasis of two important crops and how treatment with *Rhodospirillum* sp. JY3 ameliorated this negative impact. Moreover, this study elucidated the oxidative stress that may reflect the severity of HHCB treatments and the sensitivity of plant species to these HHCB, so it can be used as an indicator for the plant interaction with water pollutants introduced into the agroecosystem. Furthermore, the crop plants could detoxify HHCB *via* different metabolic strategies including augmented antioxidant defense systems to cope with the oxidative burst and improving the activities of xenobiotic metabolizing enzymes. These strategies ensure the maintenance of cellular integrity *via* manipulating plant biochemical, physiological, and molecular traits. The endpoints of these strategies can be used as biomarkers for forecasting the phytotoxic impact triggered by HHCB and similarly other water pollutants introduced to the ecosystem.

## Data availability statement

The raw data supporting the conclusions of this article will be made available by the authors, without undue reservation.

## Author contributions

Conceptualization - MM and HA; Methodology HA, SMK, and AMR; Software- AMR, SS and MM; Formal analysis - WO; Investigation - MM; Resources - HA; Data curation AMR, HA and MM; Writing the original draft – MM; Writing, reviewing and editing – MM, WO and HA; Funding acquisition SMK and WO; Validation – AMR, MM, SMK, EAA and HA; Visualization - WO and AMR. All authors contributed to the article and approved the submitted version.

## Funding

This research was funded by Princess Nourah bint Abdulrahman University Researchers Supporting Project number (PNURSP2022R214), Princess Nourah bint Abdulrahman University, Riyadh, Saudi Arabia.

## Acknowledgments

Authors are grateful for the support of Princess Nourah bint Abdulrahman University Researchers Supporting Project number (PNURSP2022R214), Princess Nourah bint Abdulrahman University, Riyadh, Saudi Arabia.

## Conflict of interest

The authors declare that the research was conducted in the absence of any commercial or financial relationships that could be construed as a potential conflict of interest.

## Publisher’s note

All claims expressed in this article are solely those of the authors and do not necessarily represent those of their affiliated organizations, or those of the publisher, the editors and the reviewers. Any product that may be evaluated in this article, or claim that may be made by its manufacturer, is not guaranteed or endorsed by the publisher.

## Supplementary material

Supplementary Figure 1
*Rhodospirillum* sp. isolate JY3 that had been used in this study. **(A)** the bacterial colonies on the plate **(B)** the microscopic examination of the isolate used in this study.
