# Supplementary material for: Rhodospirillum sp. JY3: An innovative tool to mitigate the phytotoxic impact of galaxolide on wheat (Triticum aestivum) and faba bean (Vicia faba) plants
Source: Front Plant Sci. 2022 Nov 14;13:1037474. doi: 10.3389/fpls.2022.1037474 (PMC9710512; doi:10.3389/fpls.2022.1037474)
Supplement: Supplementary Figure 1 — Rhodospirillum sp. isolate JY3 that had been used in this study. (A) the bacterial colonies on the plate (B) the microscopic examination of the isolate used in this study. [file Image_1.pdf]

Supplementary figures

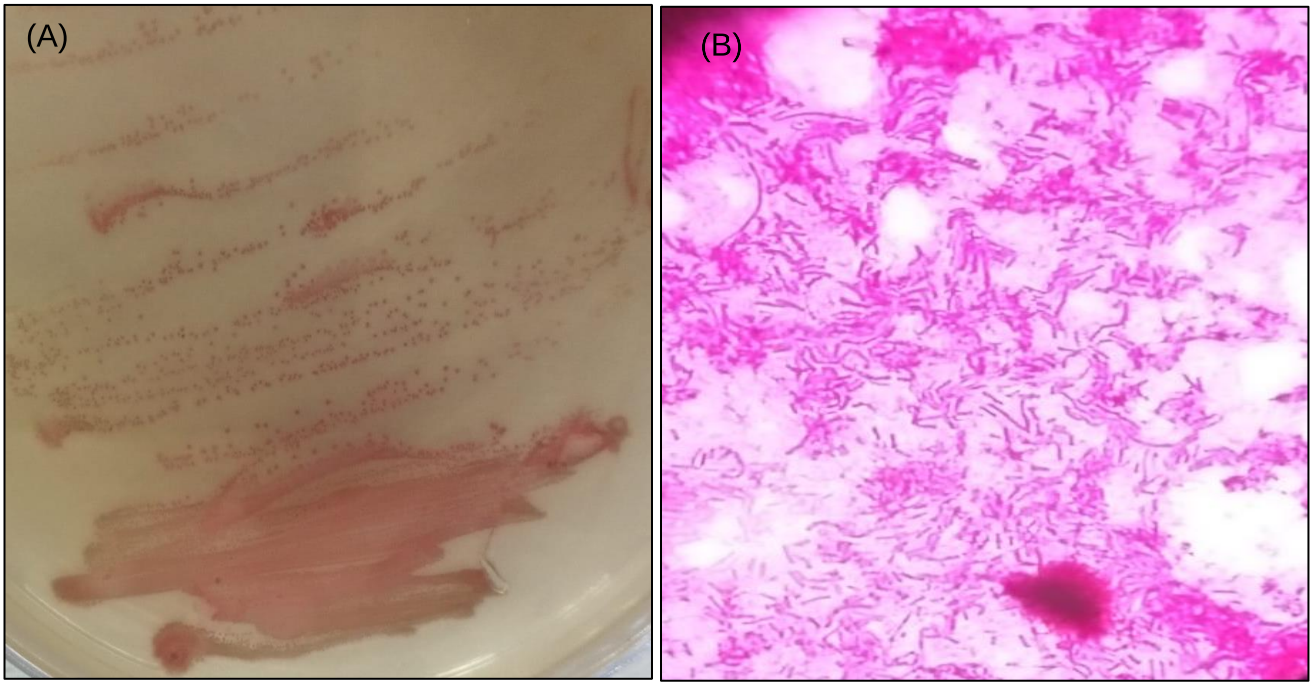

Figure S1:

Rhodospirillum sp. isolate JY3 that had been used in thus study. (A) the bacterial colonies on the plate (B) the microscopic examination of the isolate used in this study.
